# Silencing of lncRNA CHRM3-AS2 Expression Exerts Anti-Tumour Effects Against Glioma *via* Targeting microRNA-370-5p/KLF4

**DOI:** 10.3389/fonc.2022.856381

**Published:** 2022-03-11

**Authors:** Dong Wang, Qiang Chen, Jun Liu, Yuqing Liao, Qiuhua Jiang

**Affiliations:** Department of Neurosurgery, Ganzhou People’s Hospital, Ganzhou, China

**Keywords:** glioma, differentially expressed RNAs, CHRM3-AS2, miR-370-5p, KLF4

## Abstract

**Objectives:**

Long non-coding RNAs (lncRNAs) are key regulators involved in the progression of glioma, and many functional lncRNAs are yet to be identified. This study aimed to explore the function of CHRM3-AS2, a rarely reported lncRNA, in glioma, as well as the underlying mechanisms involving miR-370-5p/KLF4.

**Methods:**

Differentially expressed RNAs (DERs) were screened from two gene expression profiles of glioblastoma (GBM). Fluorescence *in situ* hybridisation was performed to determine the subcellular localisation of CHRM3-AS2. Cell viability, colony formation, apoptosis, migration, and invasion were evaluated using cell counting kit-8, colony counts, flow cytometry, wound healing, and Transwell assays, respectively. mRNA and protein expression of specific genes were measured using quantitative real-time polymerase chain reaction and western blotting, respectively. Dual luciferase reporter gene, RNA immunoprecipitation, and RNA pull-down assays were performed to identify the target relationships. A mouse xenograft model was established for *in vivo* validation.

**Results:**

CHRM3-AS2 was screened as a prognosis-associated DER in GBM. CHRM3-AS2 expression was up-regulated in glioma cells, and CHRM3-AS2 was localised in the cytoplasm. Silencing of CHRM3-AS2 expression inhibited cell viability, colony formation, migration, and invasion and promoted apoptosis of U251 and SHG-44 cells. In addition, CHRM3-AS2 targeted miR-370-5p/KLF4 in glioma cells. The anti-tumour effect of CHRM3-AS2 silencing was weakened by miR-370-5p silencing or KLF4 overexpression. *In vivo*, silencing of CHRM3-AS2 expression inhibited tumour growth and Ki67 expression in mice. Overexpression of KLF4 also weakened the anti-tumour effect of CHRM3-AS2 silencing in mice.

**Conclusions:**

Silencing of CHRM3-AS2 expression inhibited the malignant progression of glioma by regulating miR-370-5p/KLF4 expression.

## Introduction

Glioma is a prevalent type of primary intracranial carcinoma, accounting for 81% of malignant brain tumours (1). Based on pathological features, glioma can be classified into the following subtypes: astrocytoma, oligodendroglioma, oligoastrocytoma, ependymoma, glioblastoma (GBM), and mixed tumours (2). Conventional therapeutic strategies, including surgical resection, chemotherapy, and radiotherapy, are still limited in improving the prognosis of patients with glioma (3). In addition, grade IV GBM, the most common and malignant form of glioma, is more likely to be accompanied with poor prognosis because GBM stem-like cells are resistant to conventional therapy and easily undergo recurrence (4). For GBM patients receiving current standard treatments, the median overall survival is approximately 12–18 months (5). Therefore, discovery of novel therapeutic targets for glioma is urgently needed.

Long non-coding RNAs (lncRNAs), i.e. non-coding RNAs that are > 200 nucleotides in length, play important regulatory roles in biological processes at the transcriptional, post-transcriptional, and epigenetic levels (6). lncRNAs are also implicated in the occurrence and progression of glioma in the aspects of stemness, hyperproliferation, angiogenesis, and drug resistance (7). Expression of many lncRNAs has been shown to be dysregulated in glioma, such as the up-regulation of NEAT1, HOTAIR, FOXM1-AS, H19, SOX2OT, and HCP5 expression and the down-regulation of GAS5, NBAT-1, and CASC2 expression (8). These lncRNAs exert critical regulatory roles in the development of the malignant features of glioma cells. For example, NEAT1 knockdown inhibits the proliferation of glioma cells both *in vitro* and *in vivo* (9). Up-regulation of H19 expression promotes the proliferation, migration, invasion, and angiogenesis of glioma cells (10). Overexpression of GAS5 inhibits cell proliferation, migration, and invasion and promotes the apoptosis of glioma cells (11). Despite these findings, many functional lncRNAs remain to be identified. The expression of CHRM3-AS2, an lncRNA, has been reported to be down-regulated in ovarian carcinoma (12). CHRM3-AS2 is considered to be an independent prognostic factor in the prognosis of ovarian carcinoma (12). However, the functions of CHRM3-AS2 in glioma remain unclear.

MicroRNAs (miRNAs) are small, non-coding, 18–25-nucleotide long RNAs that are closely associated with glioma tumourigenesis and development. A meta-analysis found that cell-free miRNAs, especially miR-21, -125, and -222, in cerebrospinal fluid and blood are potential non-invasive biomarkers for the early diagnosis of glioma (13). Another meta-analysis revealed that increased expression of miR-15b, -21, -148a, -196, -210, and -221 and decreased expression of miR-106a and -124 are correlated with poor outcomes in patients with glioma (14). In addition, certain miRNAs have been noted to be the therapeutic targets of glioma through regulation of malignant characteristics. These targets include miR-451 (15), -93 (16), -320a (17), -27b-3p (18), and -6869-5p (19). miR-370-5p serves as an anti-oncogene in breast cancer (20), lung cancer (21), hepatocellular carcinoma (22), and ovarian cancer (23). However, the functions of miR-370-5p in glioma and related mechanisms have not been explored.

lncRNAs are considered to be competing endogenous RNAs that can competitively bind to miRNAs, thereby regulating the expression of downstream target genes of miRNAs at the post-transcriptional level (24). To date, emerging regulatory axes involving lncRNA/miRNA/mRNA have been determined to be involved in glioma progression, such as H19/miR-138/HIF-1α (10), BCYRN1/miR-619-5p/UEDC2 (25), PVT1/miR-128-3p/GREM1 (26), and SNHG16/miR-373/EGFR (27). KLF4 is a core component of the pluripotency transcription network that is involved in the regulation of cell cycle following DNA damage (28). A recent study has proved that KLF4 can be targeted by lncRNA XIST/miR-152 in regulating stemness in GBM (29). In this study, a screened prognosis-associated differentially expressed RNA (DER), CHRM3-AS2, was analysed in glioma. Then, the action mechanisms of CHRM3-AS2 involving miR-370-5p/KLF4 were evaluated. Our findings may reveal potential molecular targets for the treatment of glioma.

## Methods

### Bioinformatic Analysis

A GBM gene expression profile was downloaded from The Cancer Genome Atlas. Samples of 162 tumour tissues with clinical prognostic information and 5 normal solid tissues were used as a training dataset. Another GBM gene expression profile was downloaded from the Chinese Glioma Genome Atlas. Samples of 237 tumour tissues were used as a validation dataset. lncRNAs and mRNAs were annotated based on HUGO Gene Nomenclature Committee recommendations (30). Immune infiltration grouping (immunity_H and immunity_L) was established *via* single sample gene set enrichment analysis (ssGSEA) using GSVA package (31). The stromalScore, immuneScore, and ESTIMATEScore were analysed using the Estimate package (32). DERs were isolated using Limma package (FDR < 0.05 and |log2FC| > 0.5) (33). Prognosis-associated DERs were isolated by univariate and multivariate Cox regression analyses using Survival package (log-rank p < 0.05) (34) and then screened using Penalized package based on Cox-Proportional Hazards model (35).

### Cell Transfection

A normal human glial cell line (HEB), and four glioma cell lines (U251, SHG-44, U87, and T98) were purchased from the American Type Culture Collection (ATCC, Manassas, VA, USA). Cells were cultured in Dulbecco’s Modified Eagle’s Medium (DMEM) containing 10% foetal bovine serum (FBS), 100 U/mL penicillin, and 100 U/mL streptomycin at 37°C with 5% CO_2_. pMKO.1-GFP vector carrying shRNA-CHRM3-AS2 (sh-CHRM3-AS2, GCCAGTTCTGCTGAGAATTAT) and corresponding empty vector (sh-NC), pcDNA3.1 vector carrying full length CHRM3-AS2/KLF4 (oe-CHRM3-AS2/KLF4) and corresponding empty vector (oe-NC), miR-370-5p inhibitor (GUAACUGCAGAGACGUGACCUG) and inhibitor NC (TAACACGTCTATACGCCCA), as well as miR-370-5p mimic (CAGGUCACGUCUCUGCAGUUAC) and mimic NC (GAUGGCAUUCGAUCAGUUCUA) were packaged into lentivirus (RiboBio, Guangzhou, China) and used for transfection. Cell transfection was performed using HighGene transfection reagent (ABclonal, Wuhan, China) following the manufacturer’s instructions.

### Fluorescence *In Situ* Hybridisation

The subcellular localisation of CHRM3-AS2 was determined using FISH. Cells were fixed with 4% paraformaldehyde for 15 min, permeabilised with 0.1% Triton X-100 for 15 min, soaked in 2 × SSC solution for 30 min, and dehydrated in a graded ethanol series (3 min in each concentration). Subsequently, cells were hybridised with 1 ug/mL of probe (CY3-GCTGACAAACTCTCTTGCCC) at 37°C overnight. After incubation with 0.4 × SSC containing 0.3% Triton X-100 for 2 min at 65°C, cells were counterstained with DAPI for 5 min in the dark. Stained cells were observed under a confocal microscope (ULTRAVIEW VOX, Perkin Elmer, Waltham, MA, USA).

### Cell Viability Assay

Cell viability was determined using Cell Counting Kit-8 (CCK-8, Beyotime, China). Transfected cells were seeded into 96-well plates and cultured for 24, 48, and 72 h. Cells in each well were then incubated with 10 µL CCK-8 solution for 2 h at 37°C. The optical density at 450 nm was detected using a microplate reader (SpectraMax M4, MolecularDevices, CA, USA).

### Colony Formation Assay

Colony formation assays were performed to measure cell proliferation. Transfected cells were seeded into 6-well plates at a density of 200 cells/well. After 7 days of culture, the colonies generated were fixed with 10% methanol for 15 min and then stained with 1% crystal violet for 20 min at 25°C. Stained colonies were observed and counted under a microscope (BX53M, Olympus, Japan).

### Cell Apoptosis Assay

Cell apoptosis was detected using the Apoptosis Detection Kit (Beyotime). Transfected cells were re-suspended in 300 μL binding buffer, then incubated with 5 µL Annexin V-FITC for 15 min, and subsequently incubated with 10 µL propidium iodide for 10 min at 25°C in the dark. The apoptosis rate was detected *via* flow cytometry (CytoFLEX S, Beckman, Miami, FL, USA) using Cell Quest software (BD Biosciences, NJ, USA).

### Wound Healing Assay

Wound healing assays were performed to measure cell migration. Transfected cells were seeded into 6-well plates and cultured overnight (to 80–90% confluence). An artificial wound (scratch) was made in each well using a pipette tip. After 24 h of culture, the wound was observed under a microscope (BX53M, Olympus). The migration rate was calculated according to the healing distance before and after wounding.

### Cell Invasion Assay

Cell invasion was detected using Transwell chambers. Transfected cells in serum-free medium were added into Matrigel-coated upper chambers, and DMEM containing 10% FBS was added into the lower chamber. After being cultured for 24 h, cells in the lower chamber were fixed with methanol for 30 min and then stained with crystal violet for 20 min. The stained cells were observed under a microscope (BX53M, Olympus) and counted in five random fields.

### Quantitative Real-Time Polymerase Chain Reaction

Total RNA was extracted from cells or tissues using TRIzol reagent (Invitrogen, CA, USA). After reverse transcription using FastKing First-strand cDNA Synthesis Mix (TiangenI, China), cDNA was used as template for qRT-PCR. Using the SYBR Green qPCR Kit (Lifeint, China), qRT-PCR was performed on an Mx3000P Real-Time PCR instrument (Stratagene, CA, USA) with the following thermocycling program: 95°C for 3 min, followed by 40 cycles of 95°C for 12 s and 62°C for 40 s. The relative expression of target genes was calculated *via* the 2^-ΔΔCt^ method. U6 was used as an internal control for miR-370-5p, and GAPDH was the internal control for CHRM3-AS2 and KLF4. The primers used in qRT-PCR are shown in **Table 1**.

**Table 1:** The primers used in qRT-PCR.

Primers	Sequences
CHRM3-AS2-F	5’-TGTTCACCACTGCACACTCA-3’
CHRM3-AS2-R	5’-CGTTGTGGGCCCGTGATAAT-3’
miR-370-5p-F	5’-ACACTCCAGCTGGGCAGGTCACGTCTCTGC-3’
miR-370-5p-R	5’-CTCAACTGGTGTCGTGGAGTCGGCAATTCAGTTGAGGTAACTGC-3’
KLF4-F	5’-CCCACATGAAGCGACTTCCC-3’
KLF4-R	5’- CAGGTCCAGGAGATCGTTGAA-3’
U6-F	5’-AAAGCAAATCATCGGACGACC-3’
U6-R	5’- GTACAACACATTGTTTCCTCGGA-3’
GAPDH-F	5’- TGTGGGCATCAATGGATTTGG-3’
GAPDH-R	5’- ACACCATGTATTCCGGGTCAAT-3’

### Western Blotting

Cell or tissue samples were lysed in RIPA Lysate (Beyotime) to extract total proteins. The protein samples were separated *via* 10% sodium dodecyl sulphate–polyacrylamide gel electrophoresis and then transferred onto polyvinylidene difluoride membranes. After blocking with 5% non-fat milk for 1 h, the membrane was incubated with anti-KLF4 antibodies (1:500, Abcam, Cambridge, UK) at 4°C for 12 h. The membrane was then incubated with horseradish peroxidase (HRP)-conjugated IgG (goat anti-rabbit, 1:2000, Abcam) for 1 h at 25°C in the dark. Protein bands were visualised using ECL reagent (Thermo Fisher Scientific, CA, USA) and images were captured using a Gel Imaging System (Tanon, China).

### Target Prediction

The miRNA targets of CHRMS-AS2 were predicted using ENCORI (https://starbase.sysu.edu.cn/index.php). Between two potential targets, miR-370-5p, an anti-oncogenic miRNA was selected for analysis. Similarly, the mRNA targets of miR-370-5p were subsequently predicted using miRDB (http://www.mirdb.org/mirdb/index.html). Among 360 targets, KLF4 (rank 11, score 94) with an important role in glioma was selected for analysis.

### Dual Luciferase Reporter Gene Assay

Target relations involving miR-370-5p and CHRM3-AS2/KLF4 were determined using DLR assays. CHRM3-AS2 and KLF4 carrying wild-type binding sites (WT-CHRM3-AS2, ugaaaaagguuGCUGUGACCUg; WT-KLF4, uccgaucaacauuuaUGACCUAa) or mutant binding sites (MT-CHRM3-AS2, ugaaaaagguuGUGCACUGGUg; MT-KLF4, uccgaucaacauuuaUCUGGUc) were integrated into dual luciferase vectors (Beyotime). U251 cells were co-transfected with WT/MUT-CHRM3-AS2/KLF4 and miR-16-5p mimic/mimic NC for 48 h. The relative luciferase activity (Firefly/Renilla luciferase) was detected using Cypridina-Firefly Luciferase Dual Assay Kit (Thermo Fisher Scientific).

### RNA Immunoprecipitation Assay

RIP assays were performed to verify the target relationship involving miR-370-5p and CHRM3-AS2. Briefly, U251 cells were lysed in RIPA lysis reagent and then incubated with anti-Ago2 or IgG-conjugated beads (Millipore, Billerica, MA, USA) at 4°C overnight. The precipitates were incubated with proteinase K for 30 min at 55°C and immunoprecipitated RNAs were then extracted using TRIzol reagent. The expression of CHRM3-AS2 and miR-370-5p was detected using qRT-PCR, as described earlier.

### RNA Pull-Down Assay

The target relationship between miR-370-5p and CHRM3-AS2 was also verified *via* RNA pull-down assays. Wild-type (cAGGUCAcgucucugcaguuac) or mutant miR-370-5p (cUCCAGUcgucucugcaguuac) were labelled with biotin-conjugated RNA (WT/MT-miR-370-5p) and transfected into U251 cells. After 48 h of transfection, cells were lysed in RIPA reagent and incubated with Nucleic Acid Compatible Streptavidin Magnetic Beads (Thermo Fisher Scientific) at 25°C for 30 min in the dark. The beads were further incubated with Biotin Elution buffer for 30 min, and the eluents were collected for RNA extraction. The expression of CHRM3-AS2 was quantified using qRT-PCR as described earlier.

### Murine Tumour Xenograft Model

Female BALB/c nude mice (4 weeks old, 14 ± 2 g, HFK Bioscience, Beijing, China) were used for the establishment of a tumour xenograft animal model. Mice were fed at 25–27°C and 45–50% relative humidity with free access to water and food. Mice were subcutaneously injected with 0.1 mL transfected U251 cells at a density of 1 × 10^7^ cells/mL (n = 6 for each group). Tumour diameters were measured every 3 days using Vernier callipers, and tumour volumes were calculated as follows: (longest diameter × shortest diameter^2^)/2. Twenty-one days after injection, mice were sacrificed by intraperitoneal injection of 150 mg/kg pentobarbital sodium. The resected tumour xenografts were weighed and then used for histological examination. All animal experiments were approved by the ethics committee of our hospital in accordance with the Guide for the Care and Use of Laboratory Animals.

### Immunohistochemistry and Immunofluorescence

The expression of Ki-67 in tumour tissues was detected *via* IHC and IF. Resected tumour tissues were fixed in 4% paraformaldehyde for 24 h, dehydrated in a graded ethanol series, embedded in paraffin, and sectioned to 5–7 μm. After dewaxing in xylene and rehydration with graded ethanol, the sections received microwave irradiation in citrate buffer at 95°C for 15 min, following which they were soaked in 3% H_2_O_2_ for 20 min. The sections were blocked with 5% goat serum for 15 min and then incubated with specific anti-Ki67 antibody (1:200, Abcam) at 4°C overnight. Subsequently, the sections were incubated with secondary antibody (HRP-IgG for IHC, 1:500; Alexa Fluor^®^ 647-IgG for IF, 1:200, Abcam) for 1 h at 25°C. For IHC, positive cells were visualised with diaminobenzidine and then observed under a microscope (BX53M, Olympus). For IF, positive cells were counterstained with DAPI and then observed under a confocal microscope (ULTRAVIEW VOX, Perkin Elmer).

### Statistical Analysis

Statistical analysis was performed using GraphPad Prism v.7 software. Data are presented in the form of mean ± standard deviation. Comparisons among different groups were performed *via* one-way or two-way analysis of variance followed by Tukey’s test. P < 0.05 was considered to be statistically significant.

## Results

### Identification of DERs in GBM Samples

**Figure 1:**
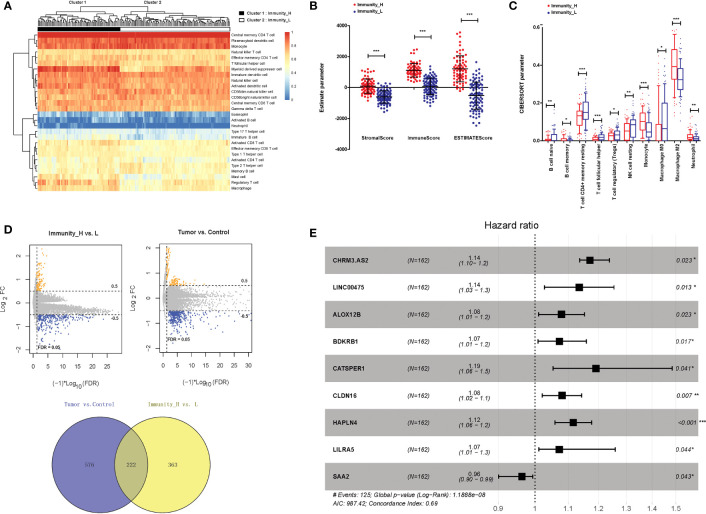
Isolation of DERs in GBM samples. **(A)** Immune infiltration grouping of GBM samples. **(B)** The stromalScore, immuneScore, and ESTIMATEScore in immunity_H and immunity_L samples. **(C)** Immune cell types in immunity_H and immunity_L samples. **(D)** DERs between immunity_H and immunity_L samples and between tumour and normal samples. **(E)** Forest plots of 9 prognosis-associated DERs (2 lncRNAs and 7 mRNAs). *P < 0.05, ** P < 0.01, ***P < 0.001.

Bioinformatics analysis was performed to determine the research objective of this study. Based on two GBM gene expression profiles, a total of 788 lncRNAs and 15,788 mRNAs were annotated. In aspect of immune infiltration, ssGSEA showed that GBM samples could be divided into two clusters, including immunity_H (72 samples) and immunity_L (90 samples) (**Figure 1A**). The stromalScore, immuneScore, and ESTIMATEScore were significantly higher in immunity_H samples than those in immunity_L samples (P < 0.001, **Figure 1B**). The immune cell types were also significantly different between immunity_H and immunity_L samples (P < 0.05, **Figure 1C**). Moreover, between immunity_H and immunity_L samples, 585 DERs were identified, and between tumour and normal samples, 798 DERs were identified. A total of 222 overlapping DERs were screened, including 12 lncRNAs and 210 mRNAs (**Figure 1D**). After being analysed *via* univariate and multivariate Cox regression analyses, 2 lncRNAs and 9 mRNAs independently associated with clinical prognosis were obtained. Furthermore, 9 DERs were confirmed *via* Cox-Proportional Hazards modelling (**Figure 1E**). CHRM3-AS2 was finally selected as the research objective for the following assays.

### CHRM3-AS2 Was Up-Regulated in Glioma Cells and Localised Cytoplasmically

**Figure 2:**
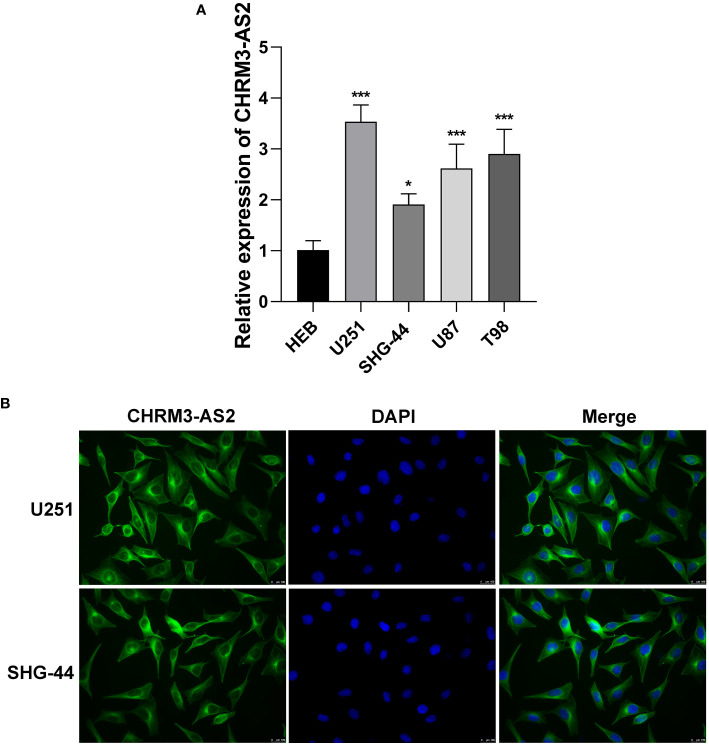
Up-regulation of expression of CHRM3-AS2 localised in the cytoplasm of glioma cells. **(A)** Expression of CHRM3-AS2 in a normal human glial cell line (HEB) and four glioma cell lines (U251, SHG-44, U87, and T98) was quantified *via* qRT-PCR. **(B)** Subcellular localisation of CHRM3-AS2 was determined *via* FISH in U251 and SHG-44 cells. ^*^P < 0.05, ^***^P < 0.001 vs. HEB.

To determine the role of CHRM3-AS2 in glioma, the expression of CHRM3-AS2 was firstly quantified in glioma cells. qRT-PCR showed that the expression of CHRM3-AS2 was significantly higher in glioma cell lines (U251, SHG-44, U87, and T98) than that in HEB cells (P < 0.05, **Figure 2A**). Subsequently, the subcellular localisation of CHRM3-AS2 in glioma cells was determined using FISH. As shown in **Figure 2B**, CHRM3-AS2 was mainly distributed in the cytoplasm of U251 and SHG-44 cells. U251 cells, with a relatively high change of CHRM3-AS2 expression, and SHG-44 cells, with relatively low change of CHRM3-AS2 expression, were selected for subsequent functional assays.

### CHRM3-AS2 Acted as an Oncogene in Glioma Cells

**Figure 3:**
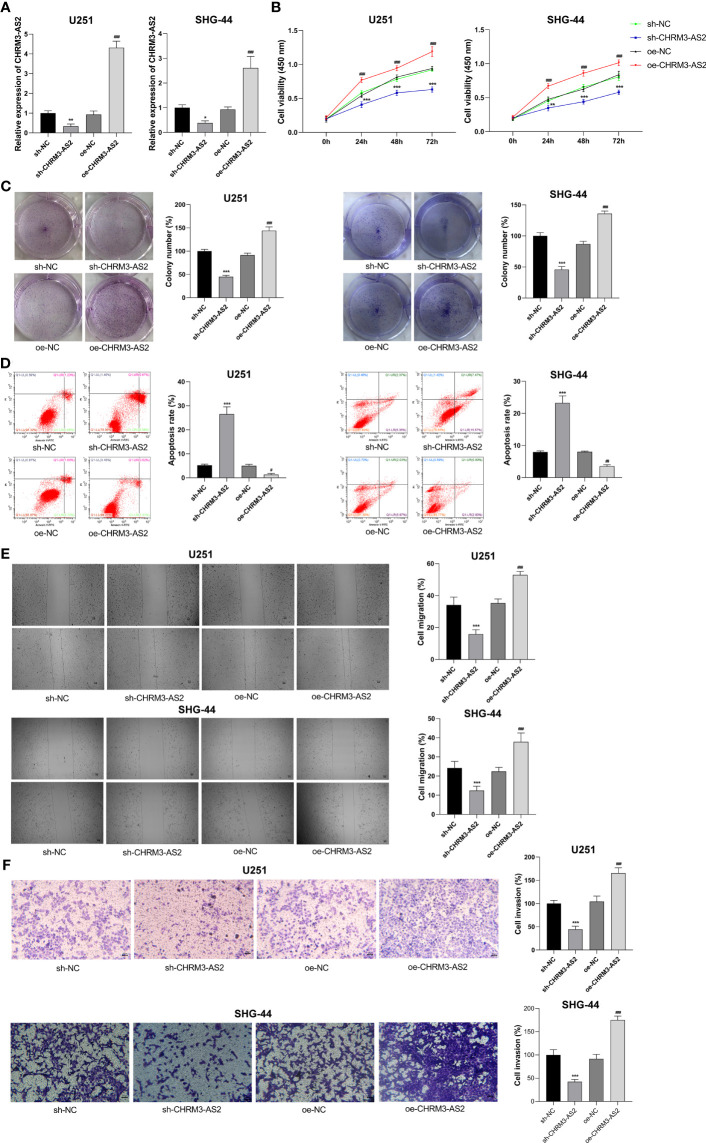
CHRM3-AS2 inhibited the malignant characteristics of glioma cells. **(A)** Expression of CHRM3-AS2 was quantified *via* qRT-PCR. **(B)** Cell viability was measured using CCK-8 assays. **(C)** Colony numbers were measured using colony formation assays. **(D)** Cell apoptosis was evaluated *via* flow cytometry. **(E)** Cell migration was evaluated using wound healing assays. **(F)** Cell invasion was analysed using Transwell assays. U251 and SHG-44 cells were transfected with sh-CHRM3-AS2/sh-NC or oe-CHRM3-AS2/oe-NC. ^*^P < 0.05, ^**^ P < 0.01, ^***^P < 0.001 vs. sh-NC; ^##^P < 0.01, ^###^P < 0.001 vs. oe-NC.

To understand the function of CHRM3-AS2 in glioma, CHRM3-AS2 was silenced or overexpressed in U251 and SHG-44 cells. qRT-PCR determined that transfection of sh-CHRM3-AS2 significantly decreased the expression of CHRM3-AS2, and transfection of oe-CHRM3-AS2 significantly increased the expression of CHRM3-AS2 in U251 and SHG-44 cells (P < 0.05, **Figure 3A**). These findings proved the effective transfection efficiency of sh-CHRM3-AS2 and oe-CHRM3-AS2 in glioma cells. As per the functional assays performed, CHRM3-AS2 silencing inhibited cell viability, colony formation, and promoted cell apoptosis in U251 and SHG-44 cells (P < 0.001, **Figures 3B–D**). The migration and invasion of U251 and SHG-44 cells were also significantly decreased by CHRM3-AS2 silencing (P < 0.001, **Figures 3E, F**). In contrast, transfection of oe-CHRM3-AS2 elicited contrary effects in U251 and SHG-44 cells, compared with transfection of sh-CHRM3-AS2 (P < 0.001, **Figures 3B–F**). The above regulatory effects of CHRM3-AS2 on the malignant characteristics of U251 and SHG-44 cells indicated an oncogenic role of CHRM3-AS2 in glioma.

### CHRM3-AS2 Regulated miR-370-5p/KLF4 in Glioma Cells

**Figure 4:**
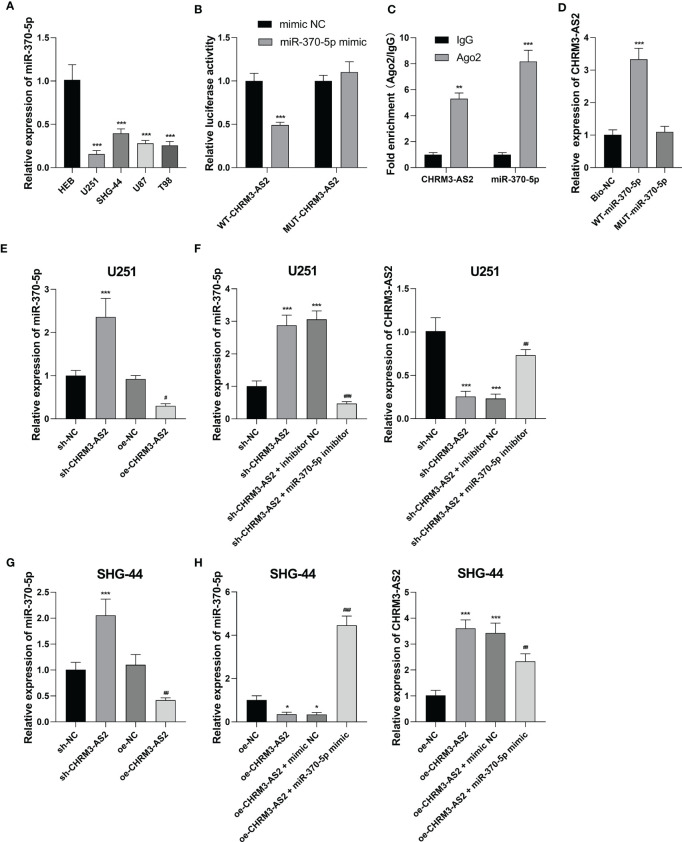
CHRM3-AS2 targeted miR-370-5p in glioma cells. **(A)** The expression of miR-370-5p in a normal human glial cell line (HEB) and four glioma cell lines (U251, SHG-44, U87, and T98) was quantified *via* qRT-PCR; ^***^P < 0.001 vs. HEB. **(B)** The relationship between CHRM3-AS2 and miR-370-5p was determined *via* DLR assays; ^***^P < 0.001 vs. mimic NC. **(C)** The relationship between CHRM3-AS2 and miR-370-5p was determined *via* RIP assays; ^**^P < 0.01, ^***^P < 0.001 vs. IgG. **(D)** The relationship between CHRM3-AS2 and miR-370-5p was determined *via* RNA pull-down assays; ^***^P < 0.001 vs. Bio-NC. **(E)** Expression of miR-370-5p in U251 cells transfected with sh-CHRM3-AS2/sh-NC or oe-CHRM3-AS2/oe-NC was quantified *via* qRT-PCR; ^***^P < 0.001 vs. sh-NC; ^#^P < 0.05 vs. oe-NC. **(F)** Expression of miR-370-5p and CHRM3-AS2 in U251 cells transfected with sh-CHRM3-AS2/sh-NC and miR-370-5p inhibitor/inhibitor NC was quantified *via* qRT-PCR; ^***^P < 0.001 vs. sh-NC; ^##^P < 0.01, ^###^P < 0.001 vs. sh-CHRM3-AS2. **(G)** Expression of miR-370-5p in SHG-44 cells transfected with sh-CHRM3-AS2/sh-NC or oe-CHRM3-AS2/oe-NC was quantified *via* qRT-PCR; ^***^P < 0.001 vs. sh-NC; ^##^P < 0.01 vs. oe-NC. **(H)** Expression of miR-370-5p and CHRM3-AS2 in SHG-44 cells transfected with oe-CHRM3-AS2/oe-NC and miR-370-5p mimic/mimic NC was quantified *via* qRT-PCR; ^*^P < 0.05, ^***^P < 0.001 vs. oe-NC; ^#^P < 0.05, ^##^P < 0.01, ^###^P < 0.001 vs. oe-CHRM3-AS2.

To understand the mechanism of action of CHRM3-AS2 in glioma, the miRNA targets of CHRM3-AS2 were predicted using Starbase 3.0. MiR-370-5p, a potential miRNA target of CHRM3-AS2, was selected for analysis. qRT-PCR showed that the expression of miR-370-5p was significantly lower in glioma cells (U251, SHG-44, U87, and T98) than that in HEB cells (P < 0.001, **Figure 4A**). The target relationship between CHRM3-AS2 and miR-370-5p was further identified by DLR, RIP and RNA pull-down assays. DLR assay showed that miR-370-5p mimic significantly decreased the luciferases activity in U251 cells transfected with WT-CHRM3-AS2 (P < 0.001), but not in cells transfected with MUT-CHRM3-AS2 (**Figure 4B**). RIP assay showed that CHRM3-AS2 was immunoprecipitated with miR-370-5p in U251 cells (P< 0.01, **Figure 4C**). RNA pull-down assay further determined that CHRM3-AS2 was pulled down by WT-miR-370-5p, but not by MUT-miR-370-5p (P< 0.001, **Figure 4D**). In addition, silencing of CHRM3-AS2 expression significantly up-regulated miR-370-5p expression (P < 0.001) and overexpression of CHRM3-AS2 significantly down-regulated miR-370-5p expression in U251 and SHG-44 cells (P < 0.05, **Figures 4E, G**). These findings indicated that CHRM3-AS2 negatively regulated its target miR-370-5p in glioma cells. Furthermore, the use of miR-370-5p inhibitor reversed the effects of sh-CHRM3-AS2 transfection (i.e. down-regulation of CHRM3-AS2 expression and up-regulation of miR-370-5p expression) (P < 0.01, **Figure 4F**). The use of miR-370-5p mimic reversed the effects of oe-CHRM3-AS2 transfection (i.e. up-regulation of CHRM3-AS2 expression and down-regulation of miR-370-5p expression) (P < 0.01, **Figure 4H**). These results further indicated that miR-370-5p could also reversely regulate CHRM3-AS2.

**Figure 5:**
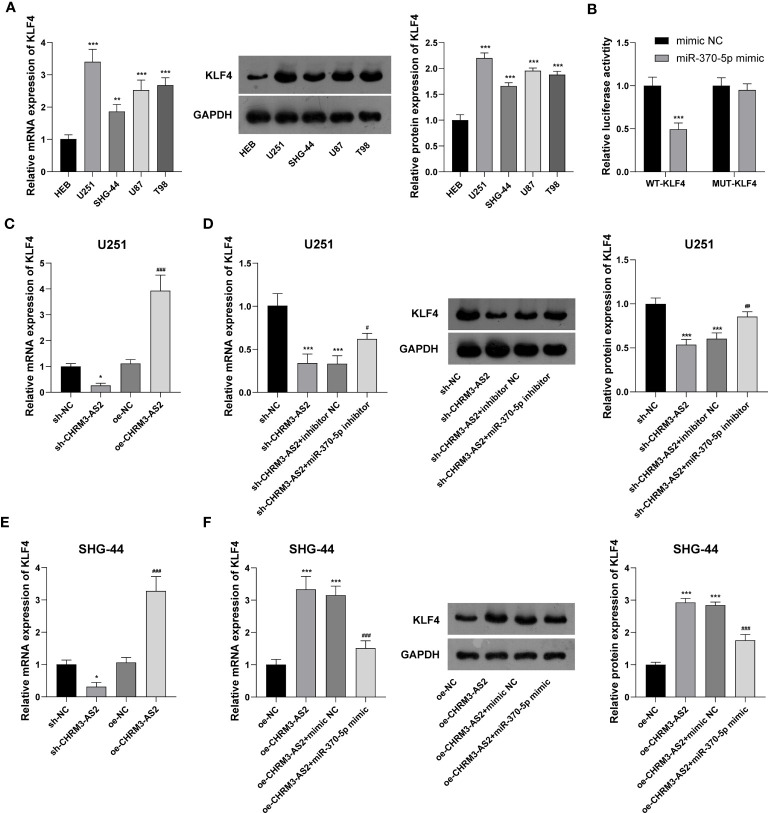
CHRM3-AS2 targeted miR-370-5p/KLF4 axis in glioma cells. **(A)** KLF4 mRNA and protein expression levels in a normal human glial cell line (HEB) and four glioma cell lines (U251, SHG-44, U87, and T98) were quantified *via* qRT-PCR and western blotting, respectively. ^**^P < 0.01, ^***^P < 0.001 vs. HEB. **(B)** The relationship between miR-370-5p and KLF4 was determined *via* DLR assays; ^***^P < 0.001 vs. mimic NC. **(C)** KLF4 mRNA expression in U251 cells transfected with sh-CHRM3-AS2/sh-NC or oe-CHRM3-AS2/oe-NC was quantified *via* qRT-PCR; ^*^P < 0.05 vs. sh-NC; ^###^P < 0.001 vs. oe-NC. **(D)** KLF4 mRNA and protein expression in U251 cells transfected with sh-CHRM3-AS2/sh-NC and miR-370-5p inhibitor/inhibitor NC was quantified *via* qRT-PCR and western blotting, respectively. ^***^P < 0.001 vs. sh-NC; ^#^P < 0.05, ^##^P < 0.01 vs. sh-CHRM3-AS2. **(E)** KLF4 mRNA in SHG-44 cells transfected with sh-CHRM3-AS2/sh-NC or oe-CHRM3-AS2/oe-NC was quantified *via* qRT-PCR; ^*^P < 0.05 vs. sh-NC; ^###^P < 0.001 vs. oe-NC. **(F)** KLF4 mRNA and protein expression in SHG-44 cells transfected with oe-CHRM3-AS2/oe-NC and miR-370-5p mimic/mimic NC was quantified *via* qRT-PCR and western blotting, respectively. ^***^P < 0.001 vs. oe-NC; ^###^P < 0.001 vs. oe-CHRM3-AS2.

Subsequently, the mRNA targets of miR-370-5p were predicted using TargetScan 7.1. KLF4, a potential mRNA target of miR-370-5p, was selected for analysis. Compared with HEB cells, glioma cells (U251, SHG-44, U87, and T98) exhibited significantly higher expression levels of KLF4 at both the mRNA and protein levels (P < 0.01, **Figure 5A**). DLR assay confirmed the target relationship between miR-370-5p and KLF4, evidenced by decreased the luciferases activity in U251 cells co-transfected with miR-370-5p mimic and WT- KLF4 (P < 0.001, **Figure 5B**). In addition, the mRNA expression of KLF4 was significantly decreased by transfection of sh-CHRM3-AS2 (P < 0.05) and increased by transfection of oe-CHRM3-AS2, in U251 and SHG-44 cells (P < 0.001, **Figures 5C, E**). The use of miR-370-5p inhibitor alleviated the inhibitory effects of sh-CHRM3-AS2 on the expression of KLF4 in U251 cells (P < 0.05, **Figure 5D**). MiR-370-5p mimic weakened the promoting effects of oe-CHRM3-AS2 on KLF4 expression in SHG-44 cells (P < 0.001, **Figure 5F**). These results indicated the presence of CHRM3-AS2/miR-370-5p/KLF4 axis in glioma cells.

### CHRM3-AS2/miR-370-5p/KLF4 Axis Regulated Malignant Characteristics of Glioma Cells

**Figure 6:**
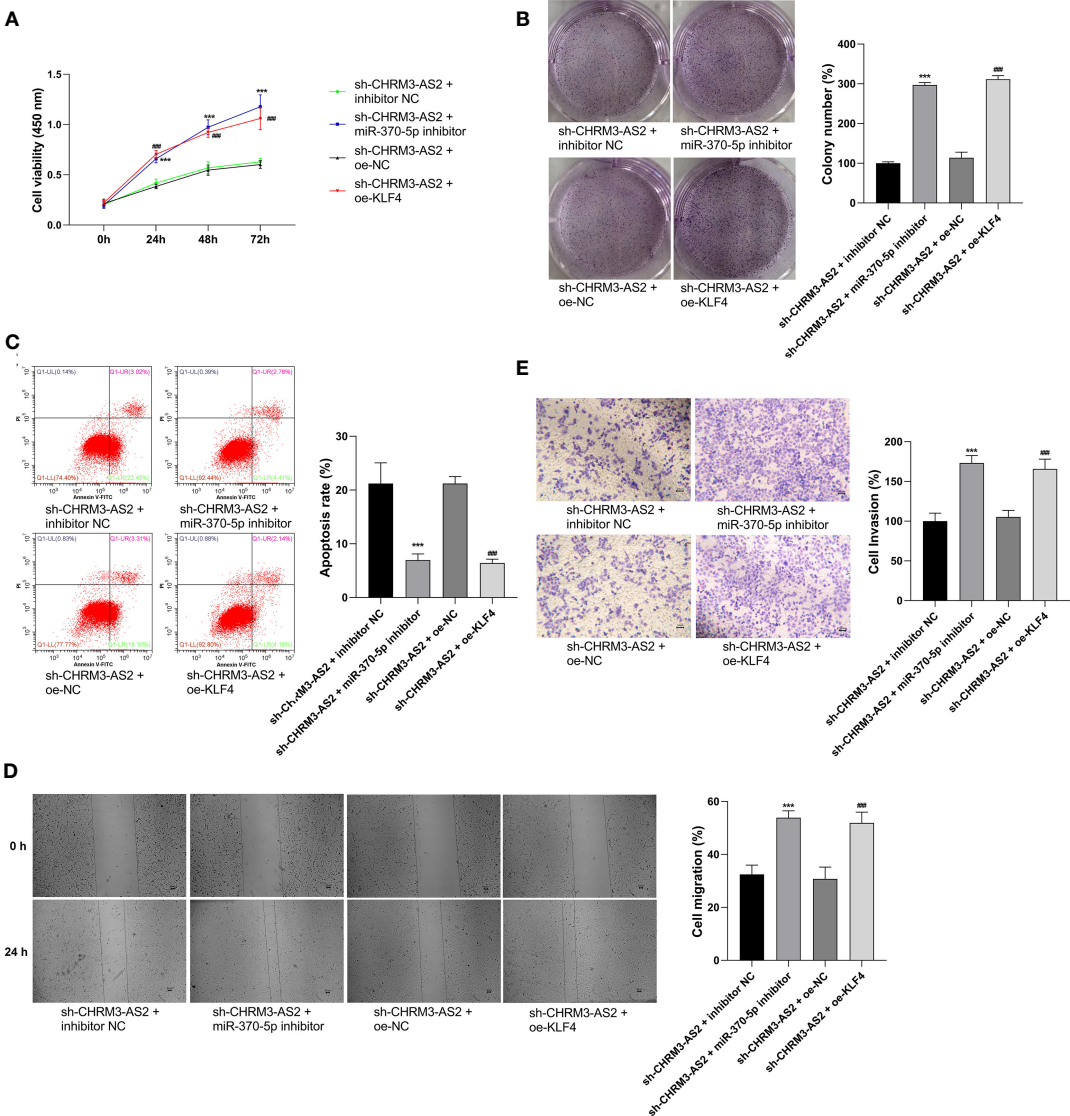
Regulatory role of CHRM3-AS2/miR-370-5p/KLF4 axis in malignant characteristics of glioma cells. **(A)** Cell viability was evaluated *via* CCK-8 assays. **(B)** Colony numbers were quantified *via* colony formation assays. **(C)** Cell apoptosis was evaluated *via* flow cytometry. **(D)** Cell migration was evaluated *via* wound healing assays. **(E)** Cell invasion was evaluated *via* Transwell assays. U251 cells were co-transfected with sh-CHRM3-AS2 and miR-370-5p inhibitor/inhibitor NC or oe-KLF4/oe-NC. ^***^P < 0.001 vs. sh-CHRM3-AS2 + inhibitor NC; ^###^P < 0.001 vs. sh-CHRM3-AS2 + oe-NC.

In view of the discovery of CHRM3-AS2/miR-370-5p/KLF4 axis in glioma cells, we suspected that CHRM3-AS2 silencing may exert inhibitory effects on glioma cells through regulating miR-370-5p/KLF4. As expected, the transfection of miR-370-5p inhibitor or oe-KLF4 significantly increased cell viability and colony formation, and decreased cell apoptosis, in sh-CHRM3-AS2-transfected U251 cells (P < 0.001, **Figures 6A–C**). In addition, the migration and invasion of sh-CHRM3-AS2-transfected U251 cells were significantly promoted by miR-370-5p silencing or KLF4 overexpression (P < 0.001, **Figures 6D, E**). These results further illustrated the regulatory role of CHRM3-AS2/miR-370-5p/KLF4 axis in glioma cells.

### Silencing of CHRM3-AS2 Inhibited Growth of Tumour Xenografts in Mice *via* Regulation of miR-370-5p/KLF4

**Figure 7:**
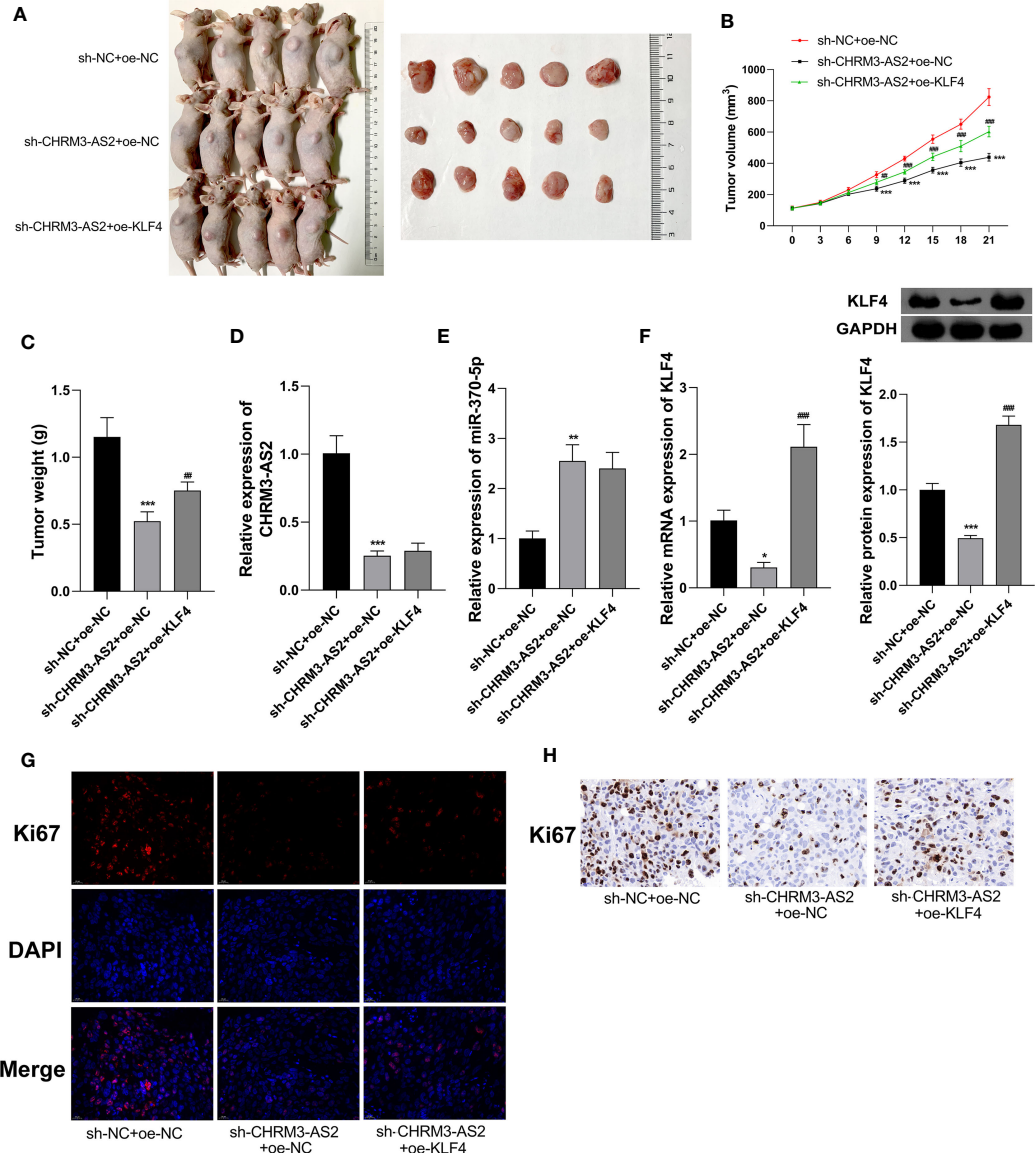
Silencing of CHRM3-AS2 expression inhibited tumour growth in a mouse tumour xenograft model by regulating miR-370-5p/KLF4 axis. **(A)** Tumour morphology. **(B)** Tumour volume. **(C)** Tumour weight. **(D)** Expression of CHRM3-AS2 was quantified *via* qRT-PCR. **(E)** Expression of miR-370-5p was quantified *via* qRT-PCR. **(F)** The mRNA and protein expression of KLF4 was quantified *via* qRT-PCR and western blotting, respectively. **(G)** Expression of Ki67 was detected *via* IF. **(H)** Expression of Ki67 was detected *via* IHC. Mice were injected with U251 cells co-transfected with sh-CHRM3-AS2/sh-NC and oe-KLF4/oe-NC. ^*^P < 0.05, ^**^P < 0.01, ^***^P < 0.001 vs. sh-NC + oe-NC; ^##^P < 0.01, ^###^P < 0.001 vs. sh-CHRM3-AS2 + oe-NC.

In addition to *in vitro* experiments, the anti-tumour potential and mechanisms of CHRM3-AS2 silencing was further investigated in a mouse tumour xenograft model *in vivo*. As shown in **Figures 7A–C**, silencing of CHRM3-AS2 expression significantly decreased tumour weights and volumes in mice (P < 0.001). The intervention of sh-CHRM3-AS2 also significantly down-regulated CHRM3-AS2 and KLF4 expression and up-regulated miR-370-5p expression in tumour xenografts (P < 0.01, **Figures 7D–F**). In addition, sh-CHRM3-AS2-induced down-regulation of KLF4 expression was reversed by the intervention of oe-KLF4 in tumour xenografts (P < 0.001, **Figure 7F**). Overexpression of KLF4 also weakened the inhibitory effects of sh-CHRM3-AS2 on tumour weight and volume (P < 0.01, **Figures 7A–C**). These results illustrated that CHRM3-AS2 silencing can inhibit tumour growth *in vivo via* regulating miR-370-5p/KLF4. Furthermore, Ki67, a robust marker of cell proliferation was detected in tumour xenografts by IF and IHC. The results showed that the expression of Ki67 was down-regulated by CHRM3-AS2 silencing in tumour xenografts, and this inhibitory effect was weakened by KLF4 overexpression (**Figures 7G, H**).

## Discussion

lncRNAs are important regulators involved in glioma tumourigenesis and progression. To date, numerous lncRNAs have been demonstrated to play tumour-inhibiting or tumour-promoting roles in glioma. Nevertheless, numerous lncRNAs are yet to be identified. In this study, a total of 12 differentially expressed lncRNAs were screened from two GBM gene expression profiles, based on tumourigenesis and immune infiltration. Furthermore, CHRM3-AS2 and LINC00475 were identified to be DERs that were independently associated with disease prognosis. Previous studies have proved that LINC00475 expression is up-regulated in glioma and can promote the progression of glioma (36, 37). As the role of CHRM3-AS2 expression in glioma is still unknown, CHRM3-AS2 was selected as the research target of this study. Similar to LINC00475, we found that CHRM3-AS2 expression was up-regulated in glioma cells. Subsequent functional assays showed that silencing of CHRM3-AS2 expression inhibited proliferation (viability and colony formation), migration, and invasion and promoted the apoptosis of glioma cells. In contrast, overexpression of CHRM3-AS2 exacerbated the malignant characteristics of glioma cells. These results indicated that CHRM3-AS2 is an oncogene in glioma, similar to NEAT1 (9), H19 (10), PVT1 (26), and DLGAP1-AS2 (38). Furthermore, silencing of CHRM3-AS2 expression inhibited the growth of tumour xenografts in mice, presenting decreased tumour weight and volume, as well as down-regulated Ki67 expression. Our findings illustrated that CHRM3-AS2 silencing is effective in inhibiting the malignant progression of glioma both *in vitro* and *in vivo*.

lncRNAs are known as endogenous sponges that can regulate the expression of specific miRNAs (39). Previous studies have proved that miR-370-5p is involved in the tumourigenesis of various tumours by acting as a target of lncRNAs such as SNHG3/miR-370-5p in colorectal carcinoma, LINC00511/miR-370-5p in ovarian cancer, and LINC01232/miR-370-5p in pancreatic adenocarcinoma. In this study, miR-370-5p was identified as a target of CHRM3-AS2 in glioma. miR-370-5p is an anti-oncogene that has been reported to be down-regulated in breast, lung, liver, and ovarian cancers (20–23). Decreased expression of miR-370-5p was consistently observed in glioma cells. In addition, studies have confirmed the anti-tumour role of miR-370-5p, with respect to the malignant characteristics of cancer cells. Sang et al. showed that overexpression of miR-370-5p suppresses the proliferation and invasion of breast cancer cells (20). Li et al. found that overexpression of miR-370-5p inhibits cell proliferation, migration, and invasion and induces cell-cycle arrest in non-small-cell lung carcinoma cells (21). Zhang et al. (22) revealed that knockdown of miR-370-5p enhances the proliferative and invasive rates of colorectal cancer cells. As miR-370-5p expression is down-regulated by CHRM3-AS2, we suspect that the anti-tumour effect of CHRM3-AS2 silencing in glioma cells is associated with the up-regulation of miR-370-5p expression. Our subsequent feedback experiments determined that the use of miR-370-5p inhibitor promoted cell proliferation, migration, and invasion and inhibited cell apoptosis in glioma cells with CHRM3-AS2 knocked down. Therefore, we conclude that silencing of CHRM3-AS2 expression inhibits the malignant progression of glioma cells *via* up-regulation of miR-370-5p expression.

As lncRNAs and miRNAs are both non-coding RNAs, their regulatory role in cancer is mainly achieved *via* modulation of target genes. To date, many miR-370-5p-associated regulatory axes have been identified in different cancers, including miR-370-5p/LUC7L3 in breast cancer (20), miR-370-5p/p21 in lung cancer (21), and SNHG3/miR-370-5p/EZH1 in colorectal carcinoma (40). In this study, KLF4 was identified as a downstream target of miR-370-5. KLF4 is a zinc finger transcription factor that functions as a tumour suppressor (in lung, gastric, and colorectal cancers) or a tumour promoter (in breast ductal carcinoma and oral/skin squamous cell carcinoma), depending on cancer type (41). KLF4 also acts as a key regulator in GBM. Wang et al. (42) have shown that KLF4 promotes spare respiratory capacity of human GBM cells by inducing mitochondrial fusion. Gong et al. revealed that SRC-1 promotes GBM stemness through KLF4 activation (29). Wang et al. found that HIF1/2α-induced KLF4 expression enhances the malignant progression of GBM by promoting stemness and cell cycle arrest (42). In this study, KLF4 was found to be up-regulated in glioma cells, indicating a tumour-promoting potential. In addition, KLF4 expression was down-regulated by miR-370-5p and up-regulated by CHRM3-AS2. Given the combination of interactions of CHRM3-AS2 and miR-370-5p with KLF4 expression, we suggest that miR-370-5p-mediated KLF4 expression is part of the underlying mechanism of action of CHRM3-AS2 on glioma cells. Furthermore, the overexpression of KLF4 was noted to weaken the anti-tumour effects of CHRM3-AS2 silencing both *in vitro* and *in vivo*. These results implied that silencing of CHRM3-AS2 expression inhibits the malignant progression of glioma by regulating the miR-370-5p/KLF4 axis.

## Conclusion

**Figure 8:**
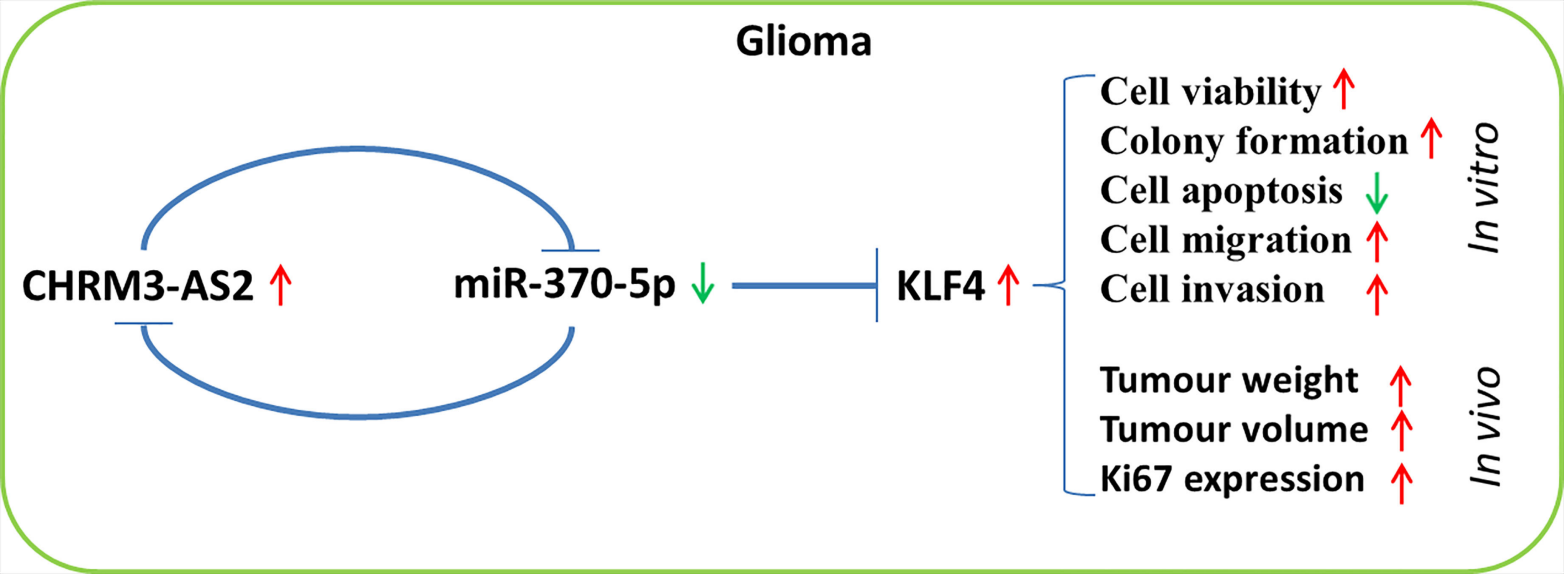
A flow chart of the regulatory axis of CHRM3-AS2/miR-370-5p/KLF4 in glioma.

In conclusion, CHRM3-AS2, a prognosis-associated DER in GBM, is an oncogene that is up-regulated in glioma cells. miR-370-5p-mediated KLF4 expression is targeted by CHRM3-AS2. Importantly, silencing of CHRM3-AS2 expression inhibited the malignant progression of glioma through regulation of the miR-370-5p/KLF4 axis. The CHRM3-AS2/miR-370-5p/KLF4 axis involved in the progression of glioma may represent a potential therapeutic target (**Figure 8**). However, the regulation of miR-370-5p/KLF4 axis is not the only action mechanism involving CHRM3-AS2 in glioma. The detailed mechanisms of action of CHRM3-AS2, as well as the clinical value of CHRM3-AS2 in glioma, require further investigation.

## Data Availability Statement

The original contributions presented in the study are included in the article/**Supplementary Material**. Further inquiries can be directed to the corresponding author.

## Ethics Statement

The animal study was reviewed and approved by the Ethics Committee of Ganzhou People’s Hospital.

## Author Contributions

DW and QJ performed the conception and design of the research. QC, JL, and YL acquired and analysed data. DW and QC draft the manuscript. DW and QJ revised the manuscript and obtained the funding. All authors contributed to the article and approved the submitted version.

## Funding

This work was supported by the Natural Science Foundation of Jiangxi Province [20181BAB205057]; the Scientific research project of Ganzhou Health Committee [2020-2-7].

## Conflict of Interest

The authors declare that the research was conducted in the absence of any commercial or financial relationships that could be construed as a potential conflict of interest.

## Publisher’s Note

All claims expressed in this article are solely those of the authors and do not necessarily represent those of their affiliated organizations, or those of the publisher, the editors and the reviewers. Any product that may be evaluated in this article, or claim that may be made by its manufacturer, is not guaranteed or endorsed by the publisher.
