# Supplementary material for: Silencing of lncRNA CHRM3-AS2 Expression Exerts Anti-Tumour Effects Against Glioma via Targeting microRNA-370-5p/KLF4
Source: Front Oncol. 2022 Mar 11;12:856381. doi: 10.3389/fonc.2022.856381 (PMC8962832; doi:10.3389/fonc.2022.856381)
Supplement: Supplementary file 1 [file DataSheet_1.docx]

The raw data for the article has been uploaded to jianguoyun, the download link is

<https://www.jianguoyun.com/p/DeYZTYgQ3rSZChii66gE>.
